# Association and Prediction of Subclinical Atherosclerosis by Nonalcoholic Fatty Liver Disease in Asymptomatic Patients

**DOI:** 10.1155/2020/8820445

**Published:** 2020-11-30

**Authors:** Ye Seul Bae, Yeon Seo Ko, Jae Moon Yun, Ah Young Eo, HaJin Kim

**Affiliations:** ^1^Department of Family Medicine, Seoul National University Hospital, Seoul, Republic of Korea; ^2^Office of Hospital Information, Seoul National University Hospital, Seoul, Republic of Korea

## Abstract

**Background:**

The prevalence of nonalcoholic fatty liver disease (NAFLD) has been increasing in the general population. This study evaluated the association between NAFLD and significant coronary stenosis in asymptomatic adults and evaluated sex-based differences.

**Methods:**

We performed a retrospective cross-sectional study in participants without previous cardiovascular diseases who visited the Seoul National University Hospital Health Promotion Center for a health checkup between January 1, 2010, and December 31, 2015. NAFLD was diagnosed on sonography, while coronary artery stenosis (CAS) was assessed on coronary computed tomography angiography (CCTA).

**Results:**

We obtained 3,693 participants who met the inclusion criteria, and 3,449 of them had no significant stenosis. Among the participants with significant stenosis, the prevalence of NAFLD was 59.4% (145 patients). The prevalence of NAFLD was 47.26% in male participants, which was higher than that in female participants. The association between NAFLD and significant CAS persisted after adjusting for age, body mass index, glycated hemoglobin, and Framingham risk factors. The correlation between NAFLD and significant coronary stenosis appeared to be stronger in women than in men, but the absolute risk was higher in men than in women.

**Conclusion:**

NAFLD was strongly associated with CAS. We should be alert about an increased cardiovascular risk in patients with NAFLD and more intensively provide primary prevention by performing tests to detect subclinical atherosclerosis.

## 1. Introduction

Cardiovascular disease (CVD) is a critical cause of death worldwide [1]. More than 25% of patients with CVD experience sudden cardiac events without prior symptoms such as chest pain or dyspnea; therefore, it is important to identify asymptomatic individuals in a subclinical state to determine when to start early prevention therapy [2].

In the current guidelines, asymptomatic adults are recommended to receive risk assessments using the Framingham coronary heart disease risk score [3], European SCORE, and atherosclerotic CVD algorithm which include several risk factors [4]. Asymptomatic subjects are generally at low or intermediate risk. In low-risk populations, the prognostic accuracy of screening tests is far from perfect, while the incorporation of coronary computed tomography angiography (CCTA) imaging data into an assessment involving the CVD risk score might improve the power of the existing tools for risk stratification. However, CCTA is associated with radiation exposure and the subsequent likelihood of radiation-induced cancer incidence. Thus, before embracing CCTA as a routine screening procedure, it should be assessed whether its potential benefits outweigh its harmful effects.

Most known risk factor assessments have included the components of metabolic syndrome (MS) [5]. Nonalcoholic fatty liver disease (NAFLD) was recently recognized as a hepatic manifestation of MS and is currently redefined as metabolic dysfunction-associated fatty liver disease (MAFLD). MAFLD indicates that this disease is associated with metabolic dysfunction, and the factors that cause and promote it are diverse [6]. While several studies have reported whether NAFLD is another independent risk factor for CVD [7–10] or systemic atherosclerosis [11, 12], previous studies have not completely excluded symptomatic adults or used a surrogate indicator such as the coronary artery calcification score [13, 14]. Other studies used carotid intima-media thickness [15], arterial stiffness, and endothelial function [16] as markers of atherosclerosis, which have limitations for directly reflecting the actual state of the coronary artery. To date, studies investigating the relationship between NAFLD and CVD have not adequately noted a tendency toward sex-based differences despite the same in NAFLD incidence and pathological mechanisms [17].

Here, we investigated the association between NAFLD and significant coronary stenosis evaluated using the CCTA in asymptomatic adults, including an additional analysis of sex-based differences.

## 2. Methods

### 2.1. Study Population

We retrospectively studied Korean adults aged >20 who visited the Seoul National University Hospital Health Promotion Center for a comprehensive health screening evaluation including abdominal sonography and CCTA between January 1, 2010, and December 31, 2015. Significant coronary stenosis was defined as the presence of >50% stenosis in at least one major coronary artery [18, 19].

We excluded those with the following criteria: history of CVDs or the use of aspirin and other antithrombotic drugs; clinical symptoms of CVD at least once such as chest pain and dyspnea (since our targeted subjects in this study had subclinical CVD); positive serology for hepatitis B virus surface antigen or hepatitis C virus antibody; history of liver cirrhosis or hepatocellular carcinoma; and excessive alcohol consumption (≥20 g/day) [20]. Furthermore, participants for whom data on study covariates were missing were also excluded.

Thus, a total of 3,693 patients were ultimately enrolled in the study. This study was approved by the Institutional Review Board of Seoul National University Hospital, Seoul, Korea, which waived the requirement for informed consent (no. H-1610-033-797).

### 2.2. Liver Ultrasound

Experienced radiologists performed hepatic ultrasonography. The diagnosis of fatty liver was based on standard criteria, including liver parenchymal brightness, evidential contrast of liver to kidney or spleen parenchyma, deep-beam attenuation, and bright vessel walls [21]. Mild NAFLD was defined as a slight increase in hepatic echogenicity and differences between hepatic and renal echogenicity as well as relative preservation of echoes from the walls of the portal vein. Moderate NAFLD was defined as a loss of echoes from the walls of the portal veins, particularly from the peripheral branches, and a greater discrepancy between the hepatic and renal echoes. Severe NAFLD was defined as a greater reduction in beam penetration, loss of echoes from most of the portal vein walls including the main branches, and a large discrepancy between the hepatic and renal echoes [22].

### 2.3. CCTA Data

CT was performed using a dual-source scanner (SOMATOM definition; Siemens Medical Solutions, Forchheim, Germany). The analytical methods of the CCTA data in this study have been described elsewhere [23]. Images were assessed by consensus to two experienced radiologists. The coronary arteries were anatomically assessed based on a modified model of the coronary tree with 15 segments. The degree of stenosis was described as the percentage of lumen diameter reduction. We then described it as significant (≥50%) or nonsignificant (<50%) based on the maximum stenosis degree [18, 19]. The plaques were defined as formed structures >1 mm^2^ within the vessel lumen and classified as noncalcified, calcified, or mixed [18, 19].

### 2.4. Clinical and Laboratory Assessment

All participants completed a personal health questionnaire and underwent a medical interview by an experienced clinician. We obtained information about past disease history (diabetes, hypertension (HTN), CVD, chronic hepatitis B, chronic hepatitis C, or malignancy), smoking status, alcohol use, medication use, and previous cardiac symptoms. The anthropometric measurements performed on the same day included height, weight, waist circumference (WC), and systolic and diastolic blood pressure. Body mass index (BMI) was calculated as weight divided by height squared, while WC was measured at the midpoint of the lowest costal margin and the iliac crest.

Blood samples for laboratory tests were taken after at least a 12-hour fast. The laboratory examination included serum levels of glucose, HbA1c, total cholesterol (TC), triglycerides, high-density lipoprotein (HDL) cholesterol, low-density lipoprotein (LDL) cholesterol, aspartate aminotransferase (AST), alanine aminotransferase (ALT), and gamma-glutamyltransferase (*γ*-GTP).

Systolic blood pressure (SBP) ≥ 140 mmHg and diastolic blood pressure (DBP) ≥ 90 mmHg in the anthropometric measurements or use of antihypertensive medications was considered as HTN [24]. Diabetes was defined as a serum glucose level ≥ 126 mg/dL, HbA1c ≥ 6.5%, or use of antidiabetic medication [25]. According to the Adult Treatment Panel III criteria, dyslipidemia was defined as TC ≥ 240 mg/dL, LDL cholesterol ≥ 190 mg/dL, or current use of dyslipidemia medication [26].

### 2.5. Statistical Analysis

Descriptive statistics were used to determine the basic characteristics of the study population. Baseline characteristics were analyzed according to significant stenosis or sex, and between-group differences were tested using *t* tests for continuous variables and the *χ*^2^ tests for categorical variables.

We used multivariate logistic regression models to estimate odds ratios with 95% confidence intervals (CIs) for significant coronary stenosis associated with the presence of NAFLD adjusted for other risk factors. We fitted two models with adjustment for potential confounders.

Model 1 was adjusted for age. Model 2 was further adjusted for BMI, HbA1c [27], and Framingham risk factors (TC, HDL cholesterol, SBP, HTN status, and smoking status) in addition to the variables addressed in Model 1. Data were analyzed using STATA for Windows version 14 (College Station, TX 77845 USA). Statistical significance was set at *P* < 0.05.

We further analyzed the general characteristics according to sex and significant stenosis. Additional analysis was conducted regarding the correlation between coronary stenosis and NAFLD among participants with normal liver function test (LFT). Normal liver function was defined as a case in which both aspartate aminotransferase (AST) and alanine aminotransferase (ALT) were less than 40 IU/L. The NAFLD fibrosis score and fibrosis-4 (FIB-4) index were measured to evaluate an invasive score in patients with NAFLD grades 2 and 3.

## 3. Results

### 3.1. Characteristics of Participants

**Figure 1:**
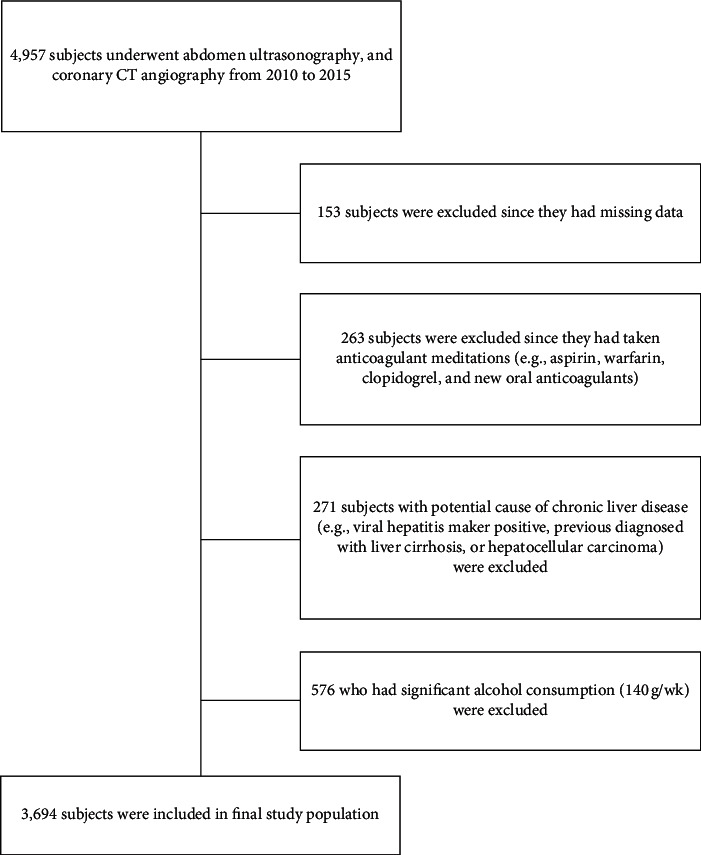
Flowchart of the included study participants.

Among 4,957 eligible patients, 3,693 patients (1,970 men and 1,723 women) met the study's inclusion criteria, while 153 patients were excluded owing to missing data. A total of 263 patients were excluded because they were on anticoagulant medications, 271 patients were excluded because they had a potential for chronic liver disease (e.g., viral hepatitis marker positivity, previously diagnosed with liver cirrhosis, or hepatocellular carcinoma), and 576 patients were excluded because of significant alcohol consumption (140 g/week) (Figure 1).

Table 1 shows the descriptive characteristics of the study population. Compared with participants without stenosis, those with significant stenosis tended to be men and smokers and had higher levels of blood pressure, fasting glucose, and triglyceride levels. Supplemental Table 1 shows the baseline characteristics according to sex. Compared with female participants, male participants tended to be overweight and smokers and had higher blood pressure, fasting glucose, TC, triglyceride, HDL cholesterol, ALT, and *γ*-GTP levels. Men showed a higher prevalence of NAFLD (47.26%, 731 patients) than women (38.19%, 658 patients). Supplemental Table 2 lists the general characteristics of the participants according to the presence or absence of significant stenosis stratified by sex. SBP, HbA1c, TG, HDL cholesterol, and the hypertension status were significant in both sexes. In contrast, BMI and WC were only significant in women.

### 3.2. Main Findings

An analysis of the relationship between NAFLD and the presence of significant stenosis is shown in Table 2. The association persisted after further adjustment for age, sex, BMI, HbA1c, and Framingham risk factors (TC, HDL cholesterol, SBP, HTN status, and smoking status) (Model 2). In this model, as shown in Table 2, NAFLD was significantly associated with CVD.

**Figure 2:**
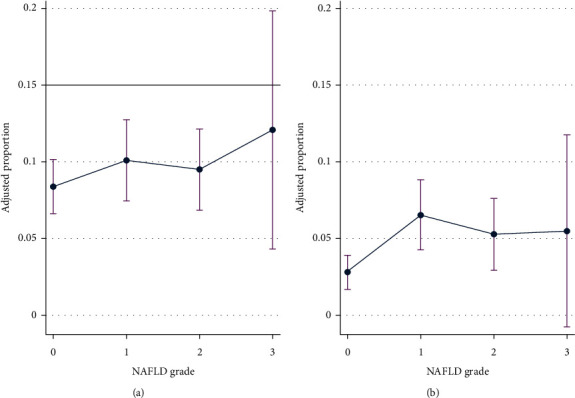
Risk ratio and predictive probability (95% CI) by degree of NAFLD in (a) male and (b) female.

We performed additional analysis to explore the association between NAFLD and the presence of significant stenosis stratified by sex (Table 3). This association was consistent with the main analysis (Model 1). However, in men, the association was attenuated after adjustment for age, BMI, HbA1c, and Framingham risk factors (*P* for trend = 0.317) (Model 2). The association between NAFLD and CVD appeared to be stronger in women (*P* for trend = 0.017) than that in men. Although the relative risk was higher in women than that in men, there was a higher absolute risk in men (Figure 2).

We explored the correlation between coronary stenosis and NAFLD among participants with normal liver function (Supplemental Table 3). The correlation was significant in the case of grade 1 NAFLD (Model 2). Supplemental Table 4 shows the NAFLD fibrosis score and the FIB-4 index in the NAFLD grade 2 and 3 groups. In the grade 2 group, 73.47% of patients had a relatively mild to moderate NAFLD fibrosis score, and 77.5% were in a stage excluded in advanced fibrosis as per the FIB-4 index. Similarly, 72.09% of patients in the grade 3 group scored relatively mild to moderate NAFLD fibrosis scores, and 74.42% were in the 0–1 fibrosis stage as per the FIB-4 index.

## 4. Discussion

Our study demonstrated that NAFLD is strongly associated with significant coronary stenosis. The correlation between NAFLD and coronary stenosis was stronger in women than that in men. There are several possible biological mechanisms of NAFLD, a hepatic manifestation of MS with insulin resistance as a common pathophysiological mechanism. NAFLD plays a critical role in the pathway connecting MS and CVD [28]. The biological mechanism by which NAFLD promotes atherosclerosis is not clear, but several previous studies have suggested a potential mechanism [12]. NAFLD may further promote insulin resistance, possibly leading to accelerated atherosclerosis [29]. In addition, possible mechanistic pathways include increased oxidative stress, subclinical inflammation, an abnormal adipocytokine profile, endothelial dysfunction, and lipid abnormalities [30].

Many studies have suggested that CVD is the leading cause of death in patients with advanced NAFLD and that NAFLD is associated with an increased risk of CVD, independent of the traditional risk factors. In addition, it is necessary to demonstrate the direct association between NAFLD and CAS to determine the starting point of cardiovascular prevention for asymptomatic patients [31]. However, most studies used arterial stiffness [16], carotid artery intima-media thickness, or carotid artery plaque measurements [15] as surrogate markers for CVD. Although some previous studies used coronary calcium score or coronary plaque [6, 32], the direct relationship between NAFLD and CAS must be assessed in the clinical setting despite the coronary calcification score being a noted marker for CVD risk [31, 33]. However, we measured the degree of CAS using the CCTA directly in asymptomatic patients.

Although there were marked sex-based differences in the prevalence of NAFLD, anthropometric and metabolic phenotypes, and adipose distribution, no other studies have demonstrated a correlation between NAFLD and coronary artery disease (CAD) according to sex. In our study, the association between NAFLD and the presence of significant stenosis was proven in women, except for those in the severe NAFLD group. Moreover, even though the relative risk was higher in women than in men, there were higher absolute risks in men. This suggests that additional underlying factors, except NAFLD, contributed to the burden of CVD risk factors in men. According to other studies, differences in major cardiovascular risk factors, particularly HDL cholesterol level and smoking rate, explained the significance of the sex difference in CAD risk [34, 35]. In women, HDL cholesterol tends to be approximately 10 mg/dL higher than that in men of the same age range [36]. Low HDL cholesterol acts as a more predictive coronary risk factor in women than in men [36, 37]. Moreover, previous studies demonstrated that women who smoke heavily lose more protection against myocardial infarction associated with HDL cholesterol than men [38, 39]. In our study, asymptomatic women had higher HDL cholesterol levels and were fewer current smokers. In addition, the significant stenosis group had a lower HDL cholesterol level than the group without significant stenosis, and this difference was statistically significant in both sexes. These findings also highlight the need for cohort studies to elucidate the role of the sex in the association between NAFLD and CVD.

In the additional analysis, the correlation between NAFLD and coronary stenosis was proven in the mild NAFLD and normal LFT groups. The mean age was 56.13 years in the moderate and severe groups and 57.80 years in the normal and mild groups. Since the participants in the moderate and severe groups were significantly younger than those in the normal and mild groups (*P* < 0.001), it is not considered that there was a significant difference in the results. The moderate to severe NAFLD but normal liver function group is not common; thus, other confounders may have an influence. To verify this, it is necessary to test the liver function at several points and conduct a study with a larger number of participants.

A notable strength of our study is its large sample size, including asymptomatic men and women. Therefore, we were able to assess each association with sex. Another strength is that CAS was directly evaluated using the CCTA. CCTA is a noninvasive and highly sensitive imaging modality that directly visualizes the coronary anatomy. In a recent study, CCTA was a valuable noninvasive tool in the diagnostic workup of patients with low to intermediate likelihood for CAS, especially in asymptomatic patients [40]. In the case of a patient diagnosed with NAFLD, there was a clinical significance; i.e., by performing CCTA, coronary stenosis was detected early in the asymptomatic stage, and an intervention was performed to slow the progression of the disease.

There are several limitations in our study. First, our study was cross-sectional in nature, making it difficult to assess causality. Second, we could not exclude any potential diseases that could influence hepatic manifestations such as autoimmune hepatic diseases owing to lack of patient information. Third, even though CCTA has emerged as a noninvasive diagnostic imaging modality that directly visualizes the coronary anatomy with a high diagnostic accuracy, the diagnosis of fatty liver depends on ultrasonography. Although ultrasonography has a relatively high sensitivity and specificity for detecting fatty liver, it may provide an incorrect diagnosis in 10%–30% of cases [41]. In addition, there are other tools such as the controlled attenuation parameter that allows to quantify steatosis in the liver, and also, using the same equipment with transient elastography, it is possible to measure liver fibrosis, an important element in patients with grade 3 steatosis [42]. Unfortunately, ultrasonography was the only device available in this study. However, since ultrasonography is a noninvasive and easily applicable clinical test, it may be a useful tool to evaluate hepatic steatosis. Future well-designed studies using sophisticated diagnostic equipment instead of abdominal sonography are required to improve the accuracy of NAFLD diagnosis.

## 5. Conclusion

Our study provides evidence for the relationship between NAFLD and significant coronary stenosis in asymptomatic patients. Accordingly, clinicians should be alert and evaluate early CVD in asymptomatic patients with NAFLD and more intensively identify primary prevention methods using tests to detect subclinical atherosclerosis.

## Figures and Tables

**Table 1:** General characteristics of the study population.

	Without significant stenosis (*n* = 3,449) (mean ± SD)	With significant stenosis (*n* = 244) (mean ± SD)	*P*value
*Sex (N, %)*			<0.001
Male	1.800 (52.18)	170 (69.67)	
Female	1.649 (47.81)	74 (30.32)	<0.001

Age (years)	57.00 ± 9.67	63.58 ± 8.69	<0.001
BMI (kg/m^2^)	24.14 ± 3.21	24.74 ± 2.87	0.004
WC (cm)	85.96 ± 9.09	88.58 ± 7.97	<0.001
SBP (mmHg)	125.40 ± 15.94	130.01 ± 15.61	<0.001
DBP (mmHg)	75.58 ± 10.65	77.07 ± 11.32	0.035
HbA1c	5.86 ± 0.68	6.26 ± 1.08	<0.001
FPG (mg/dL)	96.07 ± 20.29	109.52 ± 31.26	<0.001
TC (mg/dL)	199.56 ± 36.96	199.95 ± 37.46	0.871
TG (mg/dL)^†^	115.78 ± 67.86	138.89 ± 97.39	<0.001
HDL (mg/dL)	54.60 ± 14.47	49.26 ± 12.62	<0.001
LDL (mg/dL)	126.85 ± 34.94	129.69 ± 38.28	0.222
AST (IU/L)	25.74 ± 14.48	27.55 ± 14.16	0.059
ALT (IU/L)	27.52 ± 24.67	31.42 ± 27.49	0.017
*γ*-GTP (IU/L)	32.79 ± 33.68	38.03 ± 42.83	0.021
Hypertension (%)	1247 (36.15)	146 (59.83)	<0.001
Current smoker (%)	458 (13.27)	51 (20.90)	<0.001

*NAFLD (N, %)*			<0.001
Normal (grade 0)	2,005 (58.13)	99 (40.57)	
Mild (grade 1)	710 (20.58)	67 (27.45)	
Moderate (grade 2)	657 (19.04)	67 (27.45)	
Severe (grade 3)	76 (2.23)	11 (4.50)	

SD: standard deviation; BM: body mass index; WC: waist circumference; SBP: systolic blood pressure; DBP: diastolic blood pressure; FPG: fasting plasma glucose; TC: total cholesterol; TG: triglyceride; HDL: high-density lipoprotein cholesterol; LDL: low-density lipoprotein cholesterol; AST: aspartate aminotransferase; ALT: alanine aminotransferase; *γ*-GTP: *γ*-glutamyltranspeptidase; NAFLD: nonalcoholic fatty liver disease. ^†^Log transformation *t* test.

**Table 2:** Summary of the regression analysis of the correlation between coronary stenosis and NAFLD.

	Model 1				Model 2			
	OR (95% CI)		*P*value	*P* for trend	OR (95% CI)		*P*value	*P* for trend
*NAFLD*								
Grade 0	1	(Reference)	—	—	1	(Reference)	—	—
Grade 1	2.12	(1.53–2.97)	<0.001	<0.001	1.69	(1.19–2.41)	0.004	0.013
Grade 2	2.39	(1.71–3.35)	<0.001		1.56	(1.06–2.29)	0.023	
Grade 3	4.04	(2.23–8.11)	<0.001		2.25	(1.05–4.80)	0.037	
Age (years)	1.09	(1.07–1.10)	<0.001		1.09	(1.07–1.11)	<0.001	
Sex	0.45	(0.34–0.60)	<0.001		0.51	(0.37–0.69)	<0.001	
BMI (kg/m^2^)	—	—	—		1.00	(0.95–1.05)	0.960	
HbA1c	—	—	—		1.38	(1.21–1.58)	<0.001	
TC (mg/dL)	—	—	—		1.01	(1.00–1.01)	0.001	
HDL (mg/dL)	—	—	—		0.98	(0.97–0.99)	<0.001	
SBP (mmHg)	—	—	—		1.00	(0.99–1.01)	0.403	
Hypertension	—	—	—		2.05	(1.48–2.85)	<0.001	
Smoking status	—	—	—		1.79	(1.24–2.60)	0.002	

NAFLD: nonalcoholic fatty liver disease; OR: odds ratio; CI: confidence interval; BMI: body mass index; TC: total cholesterol; HDL: high-density lipoprotein cholesterol; SBP: systolic blood pressure. Model 1 included age and sex. Model 2 included BMI, HbA1c, and Framingham risk factors (total cholesterol, HDL cholesterol, SBP, hypertension status, and smoking status) in addition to the variables addressed in Model 1.

**Table 3:** Summary of the regression analysis of the correlation between coronary stenosis and NAFLD by sex.

		Model 1				Model 2			
		OR (95% CI)		*P*value	*P* for trend	OR (95% CI)		*P*value	*P* for trend
Men	Grade 0	1	(Reference)	—	—	1	(Reference)	—	—
	Grade 1	1.69	(1.15∼2.58)	0.011	<0.001	1.36	(0.88–2.10)	0.170	0.317
	Grade 2	1.98	(1.33∼2.96)	0.001		1.29	(0.81–2.05)	0.278	
	Grade 3	3.45	(1.52∼7.84)	0.003		2.07	(0.84–5.08)	0.112	

Women	Grade 0	1	(Reference)	—	—	1	(Reference)	—	—
	Grade 1	3.21	(1.80–5.73)	<0.001	0.001	2.61	(1.42–4.81)	0.002	0.017
	Grade 2	3.28	(1.76–6.12)	<0.001		2.27	(1.13–4.53)	0.020	
	Grade 3	5.29	(1.43–19.52)	0.012		2.66	(0.64–11.10)	0.179	

NAFLD: nonalcoholic fatty liver disease; OR: odds ratio; CI: confidence interval. Model 1 included age. Model 2 included BMI, HbA1c, and Framingham risk factors (total cholesterol, HDL cholesterol, SBP, hypertension status, and smoking status) in addition to the variables addressed in Model 1.

## Data Availability

No data used to support the findings of the study.

## Supplementary Materials

Supplementary Materials Supplemental Table 1: general characteristics according to sex. Supplemental Table 2: baseline characteristics of the study population according to sex. Supplemental Table 3: summary of the regression analysis of the correlation between coronary stenosis and NAFLD among participants with normal liver function. Supplemental Table 4: NAFLD fibrosis score and the FIB-4 index in participants with NAFLD grade 2 and 3 groups.

## References

[1] Myerburg R. J., Interian A., Mitrani R. M., Kessler K. M., Castellanos A. (1997). Frequency of sudden cardiac death and profiles of risk. *The American Journal of Cardiology*.

[2] Greenland P., Smith S. C., Grundy S. M. (2001). Improving coronary heart disease risk assessment in asymptomatic people. *Circulation*.

[3] Greenland P., Alpert J. S., Beller G. A. (2010). 2010 ACCF/AHA guideline for assessment of cardiovascular risk in asymptomatic adults. *Journal of the American College of Cardiology*.

[4] Goff D. C., Lloyd-Jones D. M., Bennett G. (2014). 2013 ACC/AHA guideline on the assessment of cardiovascular risk: a report of the American college of cardiology/American heart association task force on practice guidelines. *Circulation*.

[5] Dekker J. M., Girman C., Rhodes T. (2005). Metabolic syndrome and 10-year cardiovascular disease risk in the Hoorn Study. *Circulation*.

[6] Eslam M., Sanyal A. J., George J. (2020). MAFLD: a consensus-driven proposed nomenclature for metabolic associated fatty liver disease. *Gastroenterology*.

[7] Ampuero J., Gallego-Durán R., Romero-Gómez M. (2015). Association of NAFLD with subclinical atherosclerosis and coronary-artery disease: meta-analysis. *Revista Espanola De Enfermedades Digestivas: Organo Oficial De La Sociedad Espanola de Patologia Digestiva*.

[8] Wu S., Wu F., Ding Y., Hou J., Bi J., Zhang Z. J. (2016). Association of non-alcoholic fatty liver disease with major adverse cardiovascular events: a systematic review and meta-analysis. *Scientific Reports*.

[9] Targher G., Byrne C. D., Lonardo A., Zoppini G., Barbui C. (2016). Non-alcoholic fatty liver disease and risk of incident cardiovascular disease: a meta-analysis. *Journal of Hepatology*.

[10] Targher G., Day C. P., Bonora E. (2010). Risk of cardiovascular disease in patients with nonalcoholic fatty liver disease. *New England Journal of Medicine*.

[11] Koo B. K., Allison M. A., Criqui M. H., Denenberg J. O., Wright C. M. (2020). The association between liver fat and systemic calcified atherosclerosis. *Journal of Vascular Surgery*.

[12] Valencia-Rodríguez A., Vera-Barajas A., Barranco-Fragoso B., Kúsulas-Delint D., Qi X., Méndez-Sánchez N. (2019). New insights into the association between non-alcoholic fatty liver disease and atherosclerosis. *Annals of Translational Medicine*.

[13] Kim D., Choi S.-Y., Park E. H. (2012). Nonalcoholic fatty liver disease is associated with coronary artery calcification. *Hepatology*.

[14] Chang Y., Ryu S., Sung K.-C. (2019). Alcoholic and non-alcoholic fatty liver disease and associations with coronary artery calcification: evidence from the Kangbuk samsung health study. *Gut*.

[15] Targher G., Bertolini L., Padovani R., Zenari L., Zoppini G., Falezza G. (2004). Relation of nonalcoholic hepatic steatosis to early carotid atherosclerosis in healthy men: role of visceral fat accumulation. *Diabetes Care*.

[16] Vlachopoulos C., Manesis E., Baou K. (2010). Increased arterial stiffness and impaired endothelial function in nonalcoholic fatty liver disease: a pilot study. *American Journal of Hypertension*.

[17] Fernandes M., Ferraro A., de Azevedo R., Fagundes Neto U. (2010). Metabolic differences between male and female adolescents with non-alcoholic fatty liver disease, as detected by ultrasound. *Acta Paediatrica*.

[18] Choi E.-K., Choi S. I., Rivera J. J. (2008). Coronary computed tomography angiography as a screening tool for the detection of occult coronary artery disease in asymptomatic individuals. *Journal of the American College of Cardiology*.

[19] Leber A. W., Becker A., Knez A. (2006). Accuracy of 64-slice computed tomography to classify and quantify plaque volumes in the proximal coronary system. *Journal of the American College of Cardiology*.

[20] Choi S.-Y., Kim D., Kim H. J. (2009). The relation between non-alcoholic fatty liver disease and the risk of coronary heart disease in Koreans. *The American Journal of Gastroenterology*.

[21] Mathiesen U. L., Franzen L. E., Åselius H. (2002). Increased liver echogenicity at ultrasound examination reflects degree of steatosis but not of fibrosis in asymptomatic patients with mild/moderate abnormalities of liver transaminases. *Digestive and Liver Disease*.

[22] Saverymuttu S. H., Joseph A. E., Maxwell J. D. (1986). Ultrasound scanning in the detection of hepatic fibrosis and steatosis. *BMJ*.

[23] Moon J. H., Park E.-A., Lee W. (2011). The diagnostic accuracy, image quality and radiation dose of 64-slice dual-source CT in daily practice: a single institution’s experience. *Korean Journal of Radiology*.

[24] Korea Academy of Medical Sciences KCfDCaP (2019). *Evidence-based Guideline for Hypertension in Primary Care*.

[25] Korea Academy of Medical Sciences KCfDCaP (2019). *Evidence-based Guideline for Type 2 Diabetes in Primary Care*.

[26] Korea Academy of Medical Sciences KCfDCaP (2019). *Evidence-based Guideline for Dyslipidemia in Primary Care*.

[27] Sharma S., Malarcher A. M., Giles W. H., Myers G. (2004). Racial, ethnic and socioeconomic disparities in the clustering of cardiovascular disease risk factors. *Ethnicity & Disease*.

[28] Hamaguchi M., Kojima T., Takeda N. (2007). Nonalcoholic fatty liver disease is a novel predictor of cardiovascular disease. *World Journal of Gastroenterology*.

[29] Sinn D. H., Cho S. J., Gu S. (2016). Persistent nonalcoholic fatty liver disease increases risk for carotid atherosclerosis. *Gastroenterology*.

[30] Santoliquido A., Campli C. D., Miele L. (2005). Hepatic steatosis and vascular disease. *European Review for Medical and Pharmacological Sciences*.

[31] Wong V. W.-S., Wong G. L.-H., Yeung J. C.-L. (2016). Long-term clinical outcomes after fatty liver screening in patients undergoing coronary angiogram: a prospective cohort study. *Hepatology*.

[32] Assy N., Djibre A., Farah R., Grosovski M., Marmor A. (2010). Presence of coronary plaques in patients with nonalcoholic fatty liver disease. *Radiology*.

[33] Efe D., Fatih A. (2014). Assessment of the relationship between non-alcoholic fatty liver disease and CAD using MSCT. *Arquivos Brasileiros de Cardiologia*.

[34] Gaggini M., Morelli M., Buzzigoli E., DeFronzo R., Bugianesi E., Gastaldelli A. (2013). Non-alcoholic fatty liver disease (NAFLD) and its connection with insulin resistance, dyslipidemia, atherosclerosis and coronary heart disease. *Nutrients*.

[35] Jousilahti P., Vartiainen E., Tuomilehto J., Puska P. (1999). Sex, age, cardiovascular risk factors, and coronary heart disease. *Circulation*.

[36] Yahagi K., Davis H. R., Arbustini E., Virmani R. (2015). Sex differences in coronary artery disease: pathological observations. *Atherosclerosis*.

[37] Rich-Edwards J. W., Manson J. E., Hennekens C. H., Buring J. E. (1995). The primary prevention of coronary heart disease in women. *New England Journal of Medicine*.

[38] Njølstad I., Arnesen E., Lund-Larsen P. G. (1996). Smoking, serum lipids, blood pressure, and sex differences in myocardial infarction. A 12-year follow-up of the Finnmark study. *Circulation*.

[39] Taylor K., Carter T., Valente A., Wright A., Smith J., Matthews K. J. A. (1981). Sex differences in the relationships between obesity, alcohol consumption and cigarette smoking and serum lipid and apolipoprotein concentrations in a normal population. *Atherosclerosis*.

[40] Stefanini G. G., Windecker S. (2015). Can coronary computed tomography angiography replace invasive angiography?. *Circulation*.

[41] Hernaez R., Lazo M., Bonekamp S. (2011). Diagnostic accuracy and reliability of ultrasonography for the detection of fatty liver: a meta-analysis. *Hepatology*.

[42] Castera L., Forns X., Alberti A. (2008). Non-invasive evaluation of liver fibrosis using transient elastography. *Journal of Hepatology*.

