# Supplementary material for: Association and Prediction of Subclinical Atherosclerosis by Nonalcoholic Fatty Liver Disease in Asymptomatic Patients
Source: Can J Gastroenterol Hepatol. 2020 Nov 30;2020:8820445. doi: 10.1155/2020/8820445 (PMC7735832; doi:10.1155/2020/8820445)
Supplement: Supplementary Materials — Supplemental Table 1: general characteristics according to sex. Supplemental Table 2: baseline characteristics of the study population according to sex. Supplemental Table 3: summary of the regression analysis of the correlation between coronary stenosis and NAFLD among participants with normal liver function. Supplemental Table 4: NAFLD fibrosis score and the FIB-4 index in participants with NAFLD grade 2 and 3 groups. [file 8820445.f1.docx]

**Supplemental Table 1. General characteristics according to sex**

|  | Men | | | Women | | | P-value |
| --- | --- | --- | --- | --- | --- | --- | --- |
|  | (n=1,970) | | | (n=1,723) | | |  |
|  | mean | ± | SD | mean | ± | SD |  |
| Age (years) | 56.84 | ± | 10.23 | 58.01 | ± | 9.11 | 0.004 |
| BMI (kg/m^2^) | 24.49 | ± | 3.09 | 23.82 | ± | 3.28 | <0.001 |
| WC (cm) | 88.63 | ± | 8.44 | 83.27 | ± | 8.87 | <0.001 |
| SBP (mmHg) | 127.03 | ± | 15.18 | 124.18 | ± | 16.69 | <0.001 |
| DBP (mmHg) | 76.94 | ± | 10.67 | 74.24 | ± | 10.56 | <0.001 |
| HbA1c | 5.89 | ± | 0.78 | 5.87 | ± | 0.65 | 0.380 |
| FPG (mg/dL) | 98.54 | ± | 22.21 | 95.16 | ± | 20.42 | <0.001 |
| TC (mg/dL) | 195.92 | ± | 36.44 | 203.77 | ± | 37.19 | <0.001 |
| TG (mg/dL) ^†^ | 127.95 | ± | 78.21 | 105.11 | ± | 57.94 | <0.001 |
| HDL (mg/dL) | 50.26 | ± | 13.03 | 58.80 | ± | 14.56 | <0.001 |
| LDL (mg/dL) | 126.48 | ± | 35.17 | 127.67 | ± | 35.18 | 0.303 |
| AST (IU/L) | 26.47 | ± | 12.52 | 25.17 | ± | 16.39 | 0.006 |
| ALT (IU/L) | 30.90 | ± | 21.85 | 24.22 | ± | 27.51 | <0.001 |
| γ-GTP (IU/L) | 40.14 | ± | 33.93 | 25.14 | ± | 33.12 | <0.001 |
| Hypertension (%) | 749 |  | (38.02) | 644 |  | (37.38) | 0.687 |
| Current smoker (%) | 448 |  | (22.74) | 61 |  | (3.54) | <0.001 |
| NAFLD (%) |  |  |  |  |  |  | <0.001 |
| Normal (Grade 0) | 1039 |  | (52.74) | 1065 |  | (61.81) |  |
| Mild (Grade 1) | 409 |  | (20.76) | 368 |  | (21.36) |  |
| Moderate (Grade2) | 466 |  | (23.65) | 258 |  | (14.97) |  |
| Severe (Grade 3) | 56 |  | (2.84) | 32 |  | (1.86) |  |

***Abbreviations***: SD standard deviation; BM: body mass index; WC waist circumference; SBP systolic blood pressure; DBP diastolic blood pressure; FPG fasting plasma glucose; TC total cholesterol; TG triglyceride; HDL high-density lipoprotein cholesterol; LDL low-density lipoprotein cholesterol; AST aspartate aminotransferase; ALT alanine aminotransferase; γ-GTP γ-glutamyltranspeptidase; NAFLD Nonalcoholic fatty liver disease

^†^ log transformation t-test**Supplemental Table 2. Baseline characteristics of the study population according to sex.**

|  | Men (n=1,970) | | | | | | | Women (n=1,723) | | | | | | |
| --- | --- | --- | --- | --- | --- | --- | --- | --- | --- | --- | --- | --- | --- | --- |
|  | Without significant stenosis | | | With significant stenosis | | |  | Without significant stenosis | | | With significant stenosis | | |  |
|  | (n=1,800) | | | (n=170) | | |  | (n=1,649) | | | (n=74) | | |  |
|  | mean | ± | SD | mean | ± | SD | *P* | mean | ± | SD | mean | ± | SD | *P* |
| Age (years) | 56.31 |  | 10.21 | 62.54 |  | 8.60 | <0.001 | 57.76 |  | 8.99 | 65.97 |  | 8.45 | <0.001 |
| BMI (kg/m^2^) | 24.48 |  | 3.10 | 24.66 |  | 2.94 | 0.459 | 23.77 |  | 3.30 | 24.92 |  | 2.70 | 0.003 |
| WC (cm) | 88.57 |  | 8.44 | 89.25 |  | 8.41 | 0.315 | 83.11 |  | 8.92 | 87.02 |  | 6.66 | <0.001 |
| SBP (mmHg) | 126.81 |  | 15.01 | 129.31 |  | 16.71 | 0.040 | 123.85 |  | 16.77 | 131.63 |  | 12.68 | <0.001 |
| DBP (mmHg) | 76.85 |  | 10.53 | 77.80 |  | 12.09 | 0.268 | 74.19 |  | 10.61 | 75.40 |  | 9.18 | 0.334 |
| HbA1c | 5.86 |  | 0.73 | 6.24 |  | 1.14 | <0.001 | 5.85 |  | 0.62 | 6.30 |  | 0.94 | <0.001 |
| FPG (mg/dL) | 97.63 |  | 20.87 | 108.10 |  | 31.71 | <0.001 | 94.36 |  | 19.51 | 112.78 |  | 30.16 | <0.001 |
| TC (mg/dL) | 195.71 |  | 36.39 | 198.15 |  | 36.98 | 0.404 | 203.75 |  | 37.14 | 204.09 |  | 38.49 | 0.939 |
| TG (mg/dL) ^†^ | 126.56 |  | 75.35 | 142.74 |  | 102.92 | <0.001 | 103.99 |  | 56.32 | 130.04 |  | 83.30 | <0.001 |
| HDL (mg/dL) | 50.53 |  | 13.06 | 47.39 |  | 12.43 | 0.002 | 59.04 |  | 14.62 | 53.55 |  | 12.06 | 0.001 |
| LDL (mg/dL) | 126.14 |  | 34.93 | 130.04 |  | 37.53 | 0.167 | 127.62 |  | 34.95 | 128.90 |  | 40.21 | 0.758 |
| AST (IU/L) | 26.28 |  | 12.23 | 28.41 |  | 15.13 | 0.034 | 25.15 |  | 16.57 | 25.58 |  | 11.51 | 0.828 |
| ALT (IU/L) | 30.65 |  | 20.83 | 33.54 |  | 30.54 | 0.099 | 24.12 |  | 27.87 | 26.55 |  | 17.91 | 0.457 |
| r-GTP (IU/L) | 39.96 |  | 32.39 | 42.07 |  | 47.32 | 0.437 | 24.98 |  | 33.32 | 28.75 |  | 28.25 | 0.337 |
| Hypertension (%) | 652 |  | (36.22) | 97 |  | (57.05) | <0.001 | 595 |  | (36.08) | 49 |  | (66.22) | <0.001 |
| Current smoker (%) | 401 |  | (22.28) | 47 |  | (27.65) | 0.110 | 57 |  | (3.46) | 4 |  | (5.41) | 0.375 |
| NAFLD (%) |  |  |  |  |  |  | 0.015 |  |  |  |  |  |  | <0.001 |
| Normal (Grade 0) | 965 |  | (53.61) | 74 |  | (43.52) |  | 1040 |  | (63.06) | 25 |  | (33.78) |  |
| Mild (Grade 1) | 368 |  | (20.44) | 41 |  | (24.11) |  | 342 |  | (20.73) | 26 |  | (35.13) |  |
| Moderate (Grade2) | 419 |  | (23.27) | 47 |  | (27.64) |  | 238 |  | (14.43) | 20 |  | (27.02) |  |
| Severe (Grade 3) | 48 |  | (2.66) | 8 |  | (4.70) |  | 29 |  | (1.75) | 3 |  | (4.05) |  |

***Abbreviations***: SD standard deviation; BM: body mass index; WC waist circumference; SBP systolic blood pressure; DBP diastolic blood pressure; FPG fasting plasma glucose; TC total cholesterol; TG triglyceride; HDL high-density lipoprotein cholesterol; LDL low-density lipoprotein cholesterol; AST aspartate aminotransferase; ALT alanine aminotransferase; γ-GTP γ-glutamyltranspeptidase; NAFLD Nonalcoholic fatty liver disease

^†^ log transformation t-test

**Supplemental Table 3. Summary of the regression analysis of the correlation between coronary stenosis and NAFLD among participants with normal liver function**

|  | Model 1 | | | | | | Model 2 | | | | | |
| --- | --- | --- | --- | --- | --- | --- | --- | --- | --- | --- | --- | --- |
|  | OR | (95% CI) | | | P-value | *P for trend* | OR | (95% CI) | | | P-value | *P for trend* |
| NAFLD |  |  |  |  |  |  |  |  |  |  |  |  |
| Grade 0 | (reference) | | | | | <0.001 | (reference) | | | | | 0.021 |
| Grade 1 | 2.24 | (1.56 | - | 3.20) | <0.001 |  | 1.82 | (1.24 | - | 2.67) | 0.002 |  |
| Grade 2 | 1.93 | (1.28 | - | 2.92) | 0.002 |  | 1.31 | (0.83 | - | 2.08) | 0.248 |  |
| Grade 3 | 1.56 | (0.35 | - | 6.90) | 0.559 |  | 0.94 | (0.20 | - | 4.33) | 0.937 |  |

***Abbreviations***: NAFLD Nonalcoholic fatty liver disease; OR odds ratio; CI confidence interval

Model 1 included age and sex

Model 2 included BMI, HbA1_C_ and Framingham risk factors (total cholesterol, HDL cholesterol, SBP, hypertension status and smoking status) in addition to the variables addressed in model 1

**Supplemental Table 4. NAFLD fibrosis score and FIB-4 index in participants with NAFLD grade 2 and 3**

|  | Grade 2 | | | | | Grade 3 | | | | |
| --- | --- | --- | --- | --- | --- | --- | --- | --- | --- | --- |
|  | (n=724) | | | | | (n=88) | | | | |
|  | n | (%) | mean | ± | SD | n | (%) | mean | ± | SD |
| NAFLD fibrosis score |  |  |  |  |  |  |  |  |  |  |
| < -1.455 | 529 | (73.47) | -2.63 | ± | 0.82 | 62 | (72.09) | -2.61 | ± | 0.77 |
| -1.455 ~ 0.675 | 188 | (26.11) | -0.78 | ± | 0.52 | 24 | (27.91) | -0.69 | ± | 0.61 |
| > 0.675 | 3 | (0.23) | 1.33 | ± | 0.54 | 0 |  |  |  |  |
| FIB-4 index |  |  |  |  |  |  |  |  |  |  |
| < 1.45 | 558 | (77.50) | 0.99 | ± | 0.26 | 64 | (74.42) | 0.88 | ± | 0.27 |
| 1.45 ~ 3.25 | 155 | (21.53) | 1.86 | ± | 0.37 | 22 | (25.58) | 1.99 | ± | 0.44 |
| > 3.25 | 7 | (0.97) | 4.37 | ± | 1.17 | 0 |  |  |  |  |

***Abbreviations***: SD standard deviation; NAFLD Nonalcoholic fatty liver disease; FIB-4 Fibrosis-4
